# Influenza Virus Infects and Depletes Activated Adaptive Immune Responders

**DOI:** 10.1002/advs.202100693

**Published:** 2021-06-30

**Authors:** Caitlin D. Bohannon, Zachary Ende, Weiping Cao, Wadzanai P. Mboko, Priya Ranjan, Amrita Kumar, Margarita Mishina, Samuel Amoah, Shivaprakash Gangappa, Suresh K. Mittal, Jonathan F. Lovell, Adolfo García‐Sastre, Blaine A. Pfeifer, Bruce A. Davidson, Paul Knight, Suryaprakash Sambhara

**Affiliations:** ^1^ Influenza Division Centers for Disease Control and Prevention Atlanta GA 30329 USA; ^2^ Oak Ridge Institute for Science and Education (ORISE) CDC Fellowship Program Oak Ridge TN 37831 USA; ^3^ Department of Comparative Pathobiology and Purdue Institute for Inflammation Immunology and Infectious Disease Purdue University West Lafayette IN 47907 USA; ^4^ Department of Biomedical Engineering State University of New York at Buffalo Buffalo NY 14260 USA; ^5^ Global Health and Emerging Pathogens Institute Icahn School of Medicine at Mount Sinai New York NY 10029 USA; ^6^ Department of Microbiology Icahn School of Medicine at Mount Sinai New York NY 10029 USA; ^7^ Department of Medicine Division of Infectious Diseases Icahn School of Medicine at Mount Sinai New York NY 10029 USA; ^8^ The Tisch Cancer Institute Icahn School of Medicine at Mount Sinai New York NY 10029 USA; ^9^ Department of Chemical and Biological Engineering School of Engineering and Applied Sciences State University of New York at Buffalo Buffalo NY 14260 USA; ^10^ Department of Anesthesiology Jacobs School of Medicine and Biomedical Sciences State University of New York at Buffalo Buffalo NY 14260 USA; ^11^ Department of Pathology and Anatomical Sciences School of Medicine and Biomedical Sciences State University of New York at Buffalo Buffalo NY 14260 USA; ^12^ Research Service Veterans Administration Western New York Healthcare System Buffalo NY 14215 USA

**Keywords:** adenovirus, flowcytometry, immunosuppression, influenza, pneumococcus

## Abstract

Influenza infections cause several million cases of severe respiratory illness, hospitalizations, and hundreds of thousands of deaths globally. Secondary infections are a leading cause of influenza's high morbidity and mortality, and significantly factored into the severity of the 1918, 1968, and 2009 pandemics. Furthermore, there is an increased incidence of other respiratory infections even in vaccinated individuals during influenza season. Putative mechanisms responsible for vaccine failures against influenza as well as other respiratory infections during influenza season are investigated. Peripheral blood mononuclear cells (PBMCs) are used from influenza vaccinated individuals to assess antigen‐specific responses to influenza, measles, and varicella. The observations made in humans to a mouse model to unravel the mechanism is confirmed and extended. Infection with influenza virus suppresses an ongoing adaptive response to vaccination against influenza as well as other respiratory pathogens, i.e., Adenovirus and *Streptococcus pneumoniae* by preferentially infecting and killing activated lymphocytes which express elevated levels of sialic acid receptors. These findings propose a new mechanism for the high incidence of secondary respiratory infections due to bacteria and other viruses as well as vaccine failures to influenza and other respiratory pathogens even in immune individuals due to influenza viral infections.

## Graphical abstract

Influenza virus preferentially targets activated adaptive immune responders, likely through increased sialic acid expression on immune cell surfaces which allows for greater viral entry and cell death. This targeting reduces immune responders to influenza as well as other respiratory pathogens and can lead to decreased vaccine efficacy and increased co‐infection.

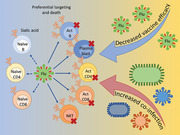

## 1 Introduction

Influenza viruses are one of the leading causes of severe illness worldwide, with ≈3 to 5 million serious cases globally, resulting in 290 000–650 000 influenza‐associated deaths annually.^[^ ^1^ ^]^ Secondary infections complicate influenza infection and add significantly to the disease burden globally. Bacterial pneumonia is the most common secondary infection – particularly *Streptococcus pneumoniae* ‐although viral coinfections, such as measles, adenovirus, or RSV, may also occur.^[^ ^2^, ^3^, ^4^, ^5^, ^6^, ^7^, ^8^, ^9^, ^10^ ^]^ Pneumoccocal infections added significantly to the mortality of the 191811–13, 1957, 1968, and 2009 influenza pandemics.^[^ ^9^, ^11^, ^12^, ^13^, ^14^, ^15^ ^]^ The observed efficacy of pneumococcal vaccination is less than 50% in patients co‐infected by influenza.^[^ ^16^ ^]^ Likewise, <40% of pneumococcal‐immunized mice co‐infected with influenza survived the lethal *S. pneumoniae* challenge.^[^ ^17^ ^]^ Influenza has evolved several strategies for immune evasion. The most well‐known of these strategies is the changing of surface glycoproteins (antigenic drift), which can escape immune detection.^[^ ^18^, ^19^ ^]^ Influenza vaccine failures can be attributed in part to a mismatch between the vaccine and circulating strains. Other potential mechanisms that have been suggested include a lack of T cell help and viral spread through intercellular nanotubes.^[^ ^20^ ^]^

Influenza virus can deplete innate responders, limit type I interferon, increase oxidative stress and cytokine storm or even damage lung tissue – all of which increase susceptibility to secondary infection.^[^ ^19^, ^21^, ^22^, ^23^, ^24^, ^25^, ^26^, ^27^, ^28^, ^29^, ^30^ ^]^ Coinfection of influenza virus and *S. pneumoniae* can also result in lowered numbers of germinal center B cells, plasma cells, and T cells in the lymph nodes and decreased antibody titers; however, this is typically attributed to the prior suppression of the innate response.^[^ ^31^ ^]^

Here, we demonstrate a mechanism by which an influenza infection directly suppresses the adaptive immune responses, preferentially infecting lymphocytes responding both to influenza and other respiratory pathogens. This targeting occurs through the increased levels of sialic acids expressed on activated lymphocytes. This results in decreased antibody‐secreting‐cells (ASCs) specific to influenza and other pathogens in both vaccinated humans and mice. Our findings indicate that influenza virus infection induces an immune‐suppressive state – even in previously vaccinated and immune individuals – by directly attacking activated immune cells, thus leaving the patients vulnerable to severe disease and leading to vaccine failures during influenza seasons.

## 2 Results

### 2.1 Influenza Virus Infection Decreases Vaccine Response in Human PBMCs

**Figure 1:**
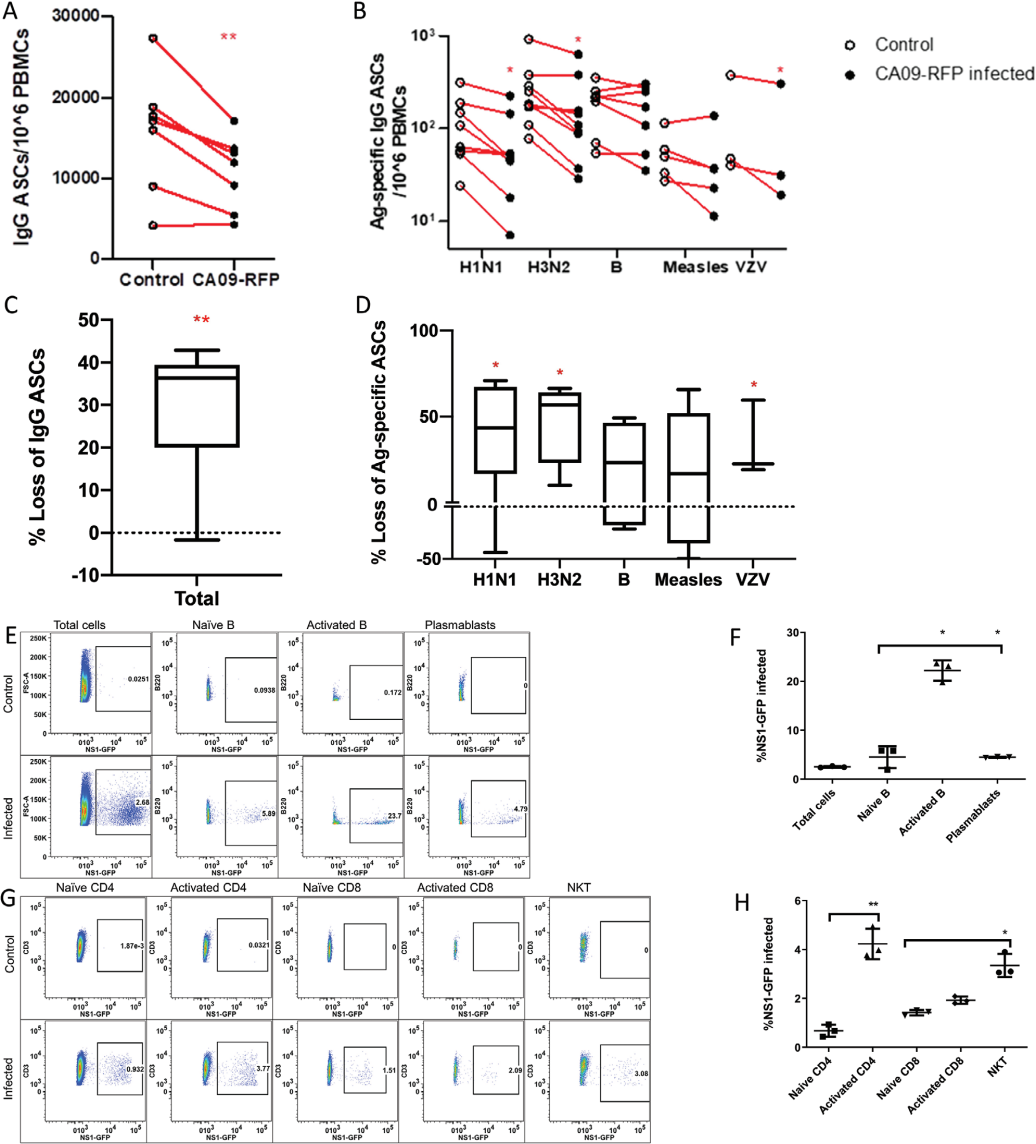
In vitro Influenza virus infection of PBMC results in decreased ASCs to H1N1, H3N2, Influenza B, and Varicella Zoster Virus (VZV) antigens, as well as total IgG. Activated lymphocytes are targeted by an influenza virus. B cell ELISPOTS from human PBMCs taken from the 2018–2019 vaccine season (*n* = 5, d28 post‐vaccination) and 2013–2014 season (*n* = 3, d7) and infected with NS1‐GFP, with A) total numbers of IgG‐expressing ASCs and B) antigen‐specific IgG ASCs in uninfected controls and infected samples are shown, matched pairs connected in red. C) Percentage loss of total IgG and D) antigen‐specific IgG for each individual, comparing paired infected and uninfected samples from each individual. The median and spread of up to 8 subjects are shown (where cells and titers were available), and (*), (**) indicating Student's paired *t*‐test *P* < 0.05, *P* < 0.01, respectively, calculated from total cell comparison (Figure 1A,B). E) Representative B cell subset flow plot highlighting percent NS1‐GFP infected for both control and day 1 p.i. PBCS, and F) bar graph showing averages across samples. G) Representative T cell subset flow plot highlighting percent NS1‐GFP infected for both control and day 1 p.i. lung cells, and H) bar graph showing averages across PBMC samples. The mean (±SD) of 3 subjects are shown, comparing Activated B cells and plasmablasts with naïve B cells, Activated CD4 with naïve CD4, and Activated CD8 and NKT cells with naïve CD8, and (*), (**) indicating Student's paired *t*‐test (where appropriate to single comparisons) or One‐way ANOVA (Dunnett's multiple comparison) of *P* < 0.05, *P* < 0.01, respectively. Total cells were excluded from statistical analysis.

Cryo‐preserved human peripheral blood mononuclear cells (PBMCs) collected from healthy adult vaccinees were treated as follows following polyclonal stimulation to increase the number of activated responders. PBMCs were infected with 5 multiplicity of infection (MOI) of CA09‐RFP (A/California/07/09 backbone; H1N1) or NS1‐GFP (A/Puerto Rico/8/34 backbone; H1N1) – which fluoresce during viral replication and the number of vaccine‐specific ASC were measured by ELISPOT (**Figure** **1**A–D). The PR8 strain was used to represent the seasonally circulating H1N1, often predominating the influenza season. The infection of PBMC by H1N1 was confirmed by flow cytometry 24 h post‐ infection (p.i.), allowing time for virus to fully replicate and trigger cell death (Figure 1E–H). A reduction (*p* < 0.01) in total IgG ASCs as well as those specific to H1N1 [*p* < 0.05 A/California/07/2009 or A/Michigan/45/2015)],H3N2 [*p* < 0.05(A/Victoria/361/2011 A/Singapore/INFIMH‐16‐ 0019/2016), B viruses (B/Massachusetts/2/2012 or B/Phuket/3073/2013), or varicella zoster virus (VZV) (*p* < 0.05) was observed. The number of measles‐specific ASCs were already low in uninfected controls, and the modest decrease did not reach statistical significance. Progressive decline in circulating measles‐specific antibodies post‐vaccination has been shown.^[^ ^32^, ^33^, ^34^, ^35^, ^36^ ^]^ Furthermore, the subjects from whom we obtained PBMC were all in the age group of 30–65. People born in 1960s through 80s used to receive one dose of measles vaccine and may have had poor memory responses to measles. Poor vaccine‐specific immunity due to one dose of vaccine and waning may have contributed to the low levels of measles‐specific ASCs observed in this study. Unlike influenza A viruses, influenza B viruses have been shown to mutate 2–3 times slower.^[^ ^37^ ^]^ Hence, B components in influenza vaccine tend to remain constant for several seasons potentially leading to reduced immunogenicity due to repeat vaccination as antigenic distance between B components between seasons is negligible. For example, B/Brisbane/60/2008like virus has been the B component of influenza vaccines since 2009–10 through 2017–18 influenza seasons. Thus, the low responses observed for influenza B virus component could be due to reduced responses owing to repeat vaccination effect. All antigen‐specificities saw a reduction with infection, with subtle differences that may be due to differing initial ASC.

### 2.2 Preferential Targeting of Activated Lymphocytes is Antigen‐Independent

Activated lymphocytes were infected at a higher proportion than naïve subsets of total PBMCs as identified by flow cytometry – specifically, activated CD4 (CD4+CD69+) and CD8 T cells (CD8+CD69+), NKTs (CD3+CD56+), activated B cells (CD19+CD69+), and plasmablasts (CD19+CD27+CD38+). However, activated CD8 T cells and plasmablasts did not reach statistical significance (Figure 1E–H, gating strategy, Figure S1: Supporting Information). In both B and T cell populations, naïve populations tended to approximate or outnumber activated populations even after stimulation, and yet active responders were disproportionately impacted.

**Figure 2:**
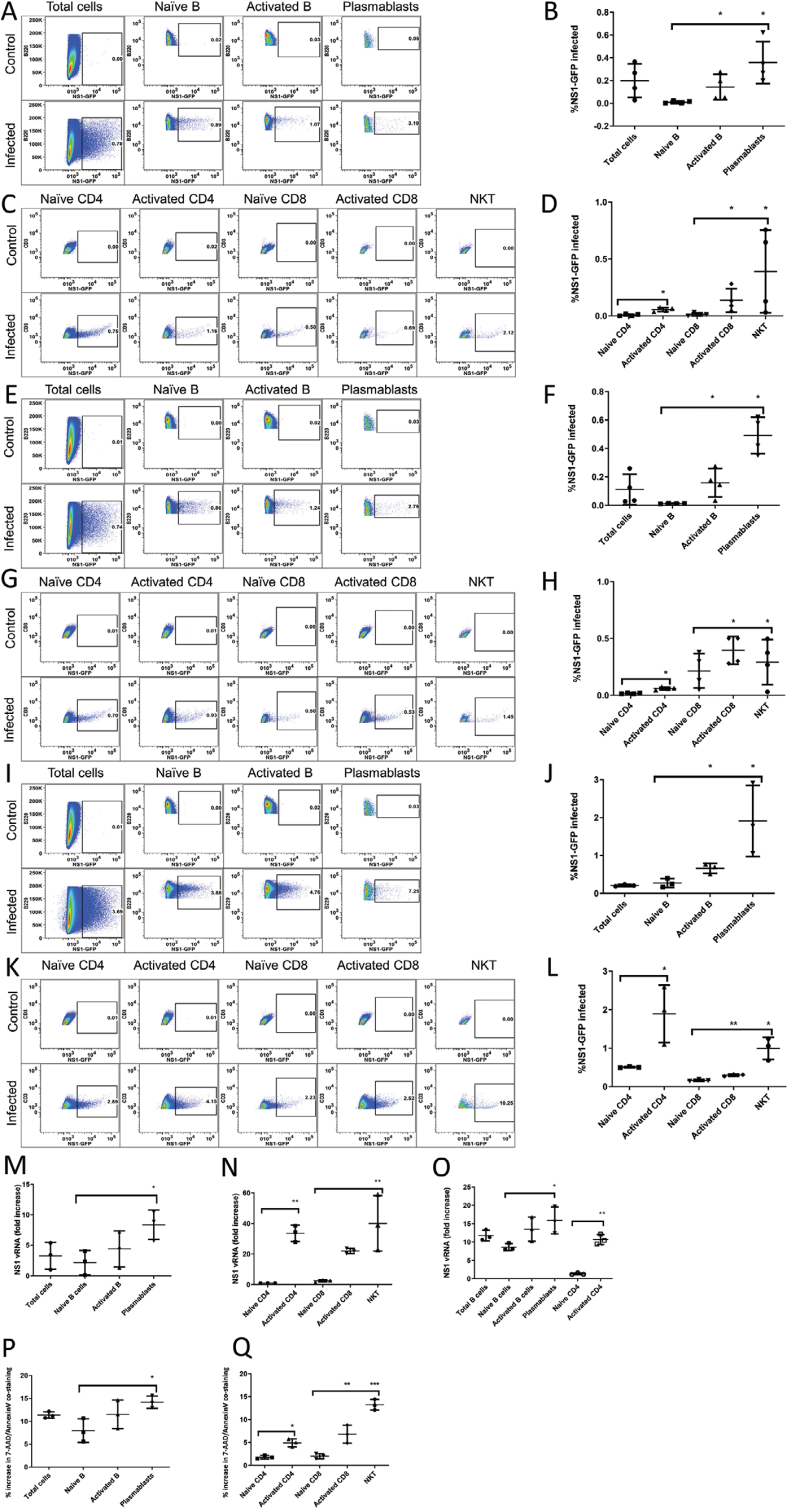
Influenza virus preferentially targets and kills activated antigen‐specific B and T cells, in vitro and in vivo. A) Representative murine B cell subset flow plot highlighting percent NS1‐GFP infected for both control and day 1 p.i. lung cells (pooled from 5 mice), isolated at day 14 post‐H1N1 immunization and boost, and B) bar graph showing averages across lung samples for 4 biological replicates of pooled samples. C) Representative murine T cell subset flow plot highlighting percent NS1‐GFP infected for both control and day 1 p.i. lung cells (pooled from 5 mice), isolated at day 14 post‐H1N1 immunization and boost, and D) bar graph showing averages across lung samples for 4 biological replicates of pooled samples. E–H) Repeated with HAd immunization and boost infected in vitro with NS1‐GFP for 24 h for 4 biological replicates of pooled samples. I–L) Repeated with HAd immunization and boost infected in vivo with NS1‐GFP for 24 h for 3 biological replicates of pooled samples. The mean (±SD) of 10 infected or uninfected lung samples are shown, comparing Activated B cells and plasmablasts with Naïve B cells, Activated CD4 with Naïve CD4, and Activated CD8 and NKT cells with Naïve CD8, and (*), (**) indicating Student's paired *t*‐test (where appropriate to single comparisons) or One‐way ANOVA (Dunnett's multiple comparison) of *P* < 0.05, *P* < 0.01, respectively. Total cells were excluded from statistical analysis. Fold increase in expression of viral NS1 gene by qPCR in sorted lung M) B and N) T cell subsets at 24 h p.i. in vitro or O) in vivo from mice previously primed with 10^9^ pfu HAd compared to uninfected controls. Sample percent apoptotic cells (compared to uninfected cells) for sorted lung P) B and Q) T subsets at 24 h post‐infection. The mean (±SD) of biological replicates of 3 pooled infected or uninfected lung samples are shown, reflecting the comparison of infected with uninfected samples, and (*), (**) indicating Student's paired *t*‐test (where appropriate) or One‐way ANOVA (Dunnett's multiple comparison) of *P* < 0.05, *P* < 0.01, respectively.

To confirm this targeting of activated lymphocytes also occurred in the lungs, BALB/c mice were immunized intranasally with 10 µg of inactivated H1N1 monovalent vaccine and boosted 7 days later, allowing for the development of adaptive responders. Lymphocytes were isolated from lungs on day 14 and infected in vitro with 5MOI of NS1‐GFP. NS1‐GFP fluorescence was measured by flowcytometry at 24 h p.i. in infected or uninfected lung lymphocytes, both from pre‐immunized mice, to ensure equivalent numbers of influenza‐ specific immune responders in both samples. Significantly (*p* < 0.01), activated B cells (CD69+) and plasmablasts (B220+CD138+), (**Figure** **2**A,B), activated CD4 and CD8 (CD62L‐CD44hi), and NKTs (CD3+DX‐5+) (Figure 2C,D), were preferentially infected by NS1‐GFP in pre‐immunized mice (gating strategy, Figure S2: Supporting Information).

To further investigate viral targeting specificity, BALB/c mouse cohorts were immunized and boosted with 10^9^ plaque‐forming units (pfu) of non‐replicating human adenovirus (HAd) intranasally before NS1‐GFP infection. Just as in lymphocytes activated by influenza vaccine, those HAd‐activated B and T cells and NKTs were similarly infected at higher levels (*p* < 0.05) than naïve lung cells (Figure 2E–H), showing that this targeting of immune responders is independent of the activating antigen. These results were further confirmed with in vivo infection of HAd‐immunized mice with 5 MOI NS1‐GFP virus, where the same pattern is observed (Figure 2I–L). As in human PBMCs, naïve B and T cells tended to approximate or outnumber active responders (particularly plasmablasts), but these populations are disproportionately infected. The observed impact appears to be lower in mice than in humans, even at the same MOI, likely due to the use of the same human NS1‐GFP virus across our experiments rather than a mouse‐adapted influenza strain.

### 2.3 Influenza Virus Infects and Lyses Activated Immune Cells

To assess the outcome of virus infection of immune responders, B‐ and T‐ cell subsets were isolated from pooled murine lungs at day 7 post‐HAd immunization and infected in vitro with 5 MOI of PR8 virus for 24 h. The levels of viral replication (determined by NS1 expression) within each subset of cells were measured by PCR, confirming viral replication in activated B and T cells and NKTs (Figure 2M,N). This was also confirmed in sorted B and T subsets isolated from lungs of mice immunized with HAd and infected at day 7 with NS1‐GFP in vivo (Figure 2O). Further, these cells were shown to increase cell apoptosis (compared to uninfected cells), measured by 7AAD and Annexin V co‐staining (Figure 2P,Q), demonstrating that viral targeting directly reduces the numbers of adaptive immune responders. This also indicated that no other external factors (such as cell‐cell contact or cytokines) are necessary for cell death in infected lymphocytes.

### 2.4 High Levels of *α*2,3 Sialic Acids Linked to Viral Entry in Mice and *α*2,6 Sialic Acids in Human PBMC

**Figure 3:**
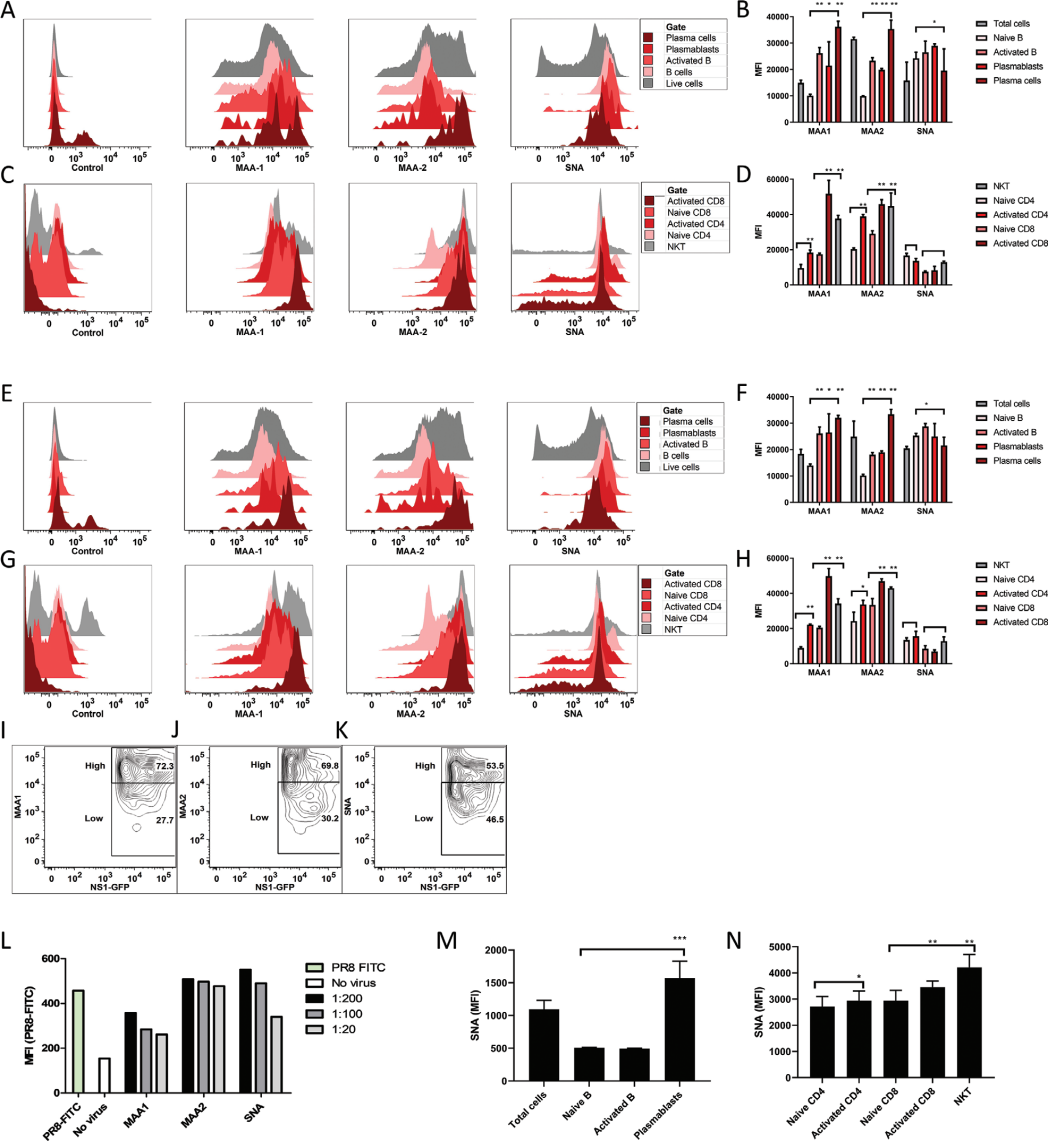
Influenza virus targets activated B and T cells through the increased expression of *α*2,3 sialic acids in mice, and *α*2,6 in humans, during a normal immune response. A) Representative murine B cell subset histograms comparing binding of MAA‐1‐, MAA‐2‐, and SNA‐FITC lectins to lung cells isolated at day 7 post‐PR8 infection, and B) bar graph showing FITC mean fluorescent intensity (MFI) averages across lung samples. C) Representative murine T cell subset histograms comparing binding of MAA‐1‐, MAA‐2, and SNA‐ FITC lectins to lung cells isolated at day 7 post‐PR8 infection, and D) bar graph showing FITC MFI averages across lung samples. Representative histograms and FITC MFI averages for murine E,F) B cell subsets and G,H) T cell subsets of lung cells isolated at day 7 post‐HAd immunization. NS1‐GFP‐infected cells were isolated and analyzed by flow cytometry for binding to I) MAA‐1, J) MAA‐2, and K) SNA, representative flow plots are shown. L) Following binding of 1:200, 1:100, 1:20, or no lectin for 30 min, levels of NS1‐GFP infection (MFI) were measured by flow cytometry in pooled murine splenocytes from 3 mice after 24 h p.i. Human PBMCs were infected with NS1‐GFP in vitro, and expression of *α*2,6, as measured by SNA‐FITC binding, is shown for M) B and N) T cell subsets. The mean (±SD.) of 3 subjects are shown, comparing activated B cells and plasmablasts with naïve B cells, activated CD4 with naïve CD4, and activated CD8 and NKT cells with naïve CD8, and (*), (**) indicating Student's paired *t*‐test (where appropriate to single comparisons) or One‐way ANOVA (Dunnett's multiple comparison) of *P* < 0.05, *P* < 0.01, respectively. Total cells were excluded from statistical analysis. All experiments were repeated at least twice with 3–4 individual biological samples.

In an earlier study of adaptive cell surface labeling, it was observed that H1N1 hemagglutinin and neuraminidase proteins bound increasingly to targeted cells [unpublished data], leading us to hypothesize that increased binding and/or viral entry might be responsible for the observed cell targeting seen here. It is well‐established that the influenza virus enters the cell via sialic acid receptors on the cell's surface. This occurs through the *α*2,6 sialic receptor in humans and the *α*2,3 in mice.^[^ ^27^ ^]^ To probe the potential mechanism of this observed viral targeting, levels of *α*2,3 and *α*2,6 sialic acids were measured at day 7 on the relevant B and T subsets following infection with 10MID_50_ of PR8 or 10^9^ pfu of HAd. Expression was measured via binding of lectins, Maackia amurensis lectin I (MAA‐1) or II (MAA‐2) to *α*2,3 sialic acids, or Sambucus nigra lectin (SNA) to *α*2,6 sialic acids. Activated B and T, as well as NKTs, revealed higher levels of *α*2,3 sialic acids compared to naïve lymphocytes in response to either PR8 (**Figure** **3**A–D) or HAd (Figure 3E–H) infection, as well as in response to immunization with Keyhole limpet hemocyanin (data not shown). Smaller differences were observed in SNA expression, along similar lines. Increases in the sialic acid expression on activated cells were observed at all‐time points and varying antigen levels (data not shown). Influenza viruses typically target *α*2,6 sialic acids in humans,^[^ ^38^ ^]^ so SNA‐binding was used to measure an increase in *α*2,6 sialic acids in activated human B and T and NKTs (Figure 3M,N), corresponding with the preferential targeting of the NS1‐GFP virus. To confirm that higher levels of *α*2,3 sialic acids in mice are directly correlated with a viral infection, lung lymphocytes – collected at day 7 post‐HAd immunization – were infected overnight with 5MOI of NS1‐GFP and then stained the following day with MAA‐1, MAA‐2, and SNA lectins and analyzed by flow cytometry. When gating on NS1‐GFP infected cells, two populations emerged – lectin(high) and lectin(low). MAA‐1 and MAA‐2(high) cells were among the majority of NS1‐GFP infected cells (>2/3 of infected cells) [representative flow plots are shown in Figure 3I,J], though this was not observed for SNA‐binders (Figure 3K). We observed a similar pattern in innate populations post‐infection. At early time points, macrophages expressed higher levels of *α*2,3 sialic acids and were preferentially infected, with a similar pattern observed in dendritic cells at later time points (Figure S3, Supporting Information).

### 2.5 Lectin Binding of Sialic Acids Blocks Viral Adhesion

To further demonstrate that the expression of sialic acids is the viral entryway, increasing amounts of lectins were added to splenocytes in vitro for 30 min before infection to show that they could block viral binding. A dose‐dependent effect was observed as increasing amounts of MAA‐1, MAA‐2, or SNA (1:200, 1:100, and 1:20 dilutions in media) were incubated with murine cells before infection with 5MOI PR8‐FITC (Figure 3L). Prior incubation of cells with 1:20 MAA‐1 lectin reduced viral adhesion to half compared to control cells. A similar, though a lesser effect, was observed with MAA‐2 and SNA.

### 2.6 In Vivo Influenza Challenge Reduces Vaccine Responses in Mice

**Figure 4:**
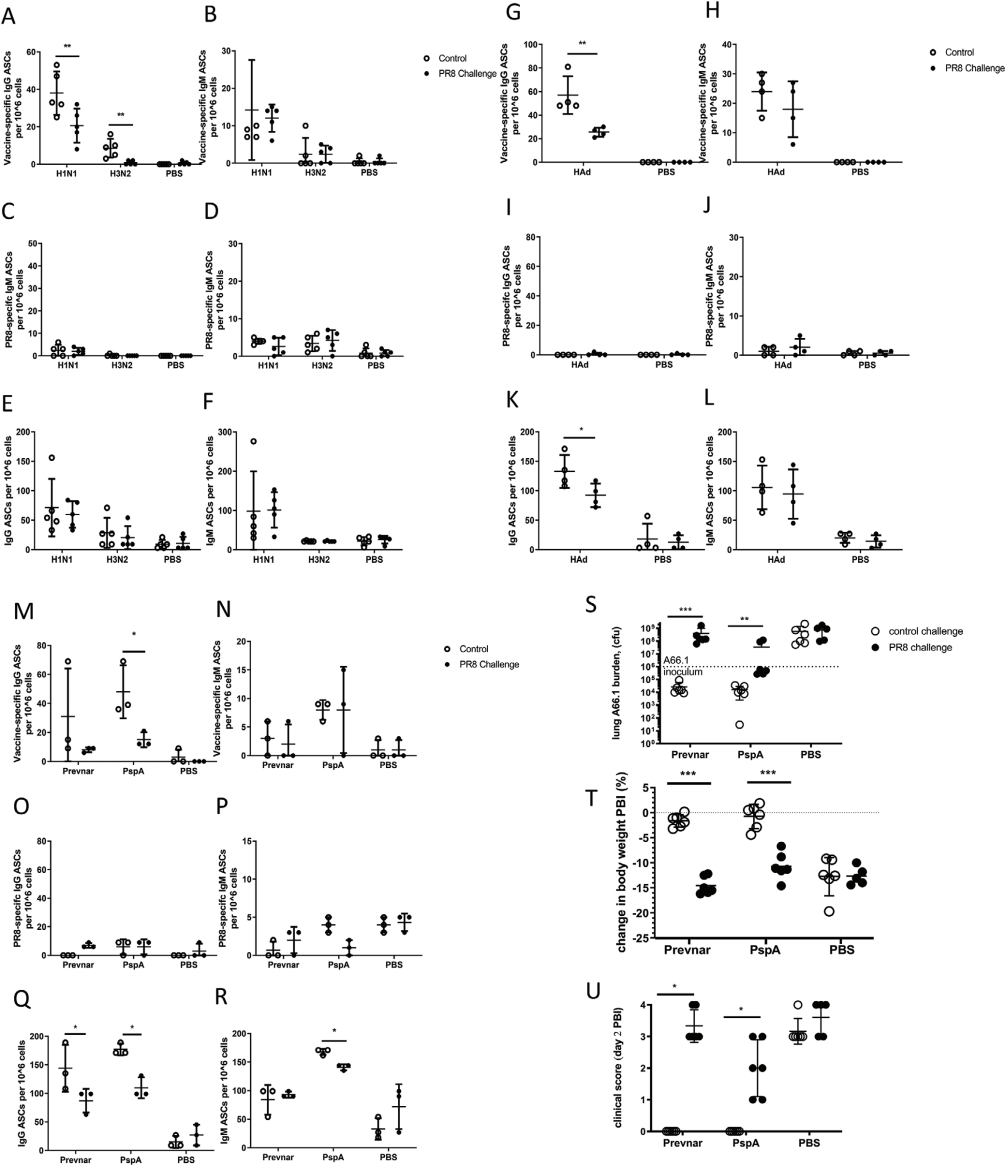
In vivo influenza challenge decreases numbers of influenza, HAd, and pneumococcal vaccine‐specific antibody‐secreting‐cells (ASCs) post‐vaccination, decreases protection against concurrent pneumococcal pulmonary challenge. Following intranasal vaccination and boost or PBS control, half of the mouse cohorts were challenged with 10MID_50_ of PR8 virus at day 14 post‐ immunization. At day 2 post‐challenge, murine lungs were isolated and analyzed by ELISpot. A) IgG and B) IgM H1N1 or H3N2‐specific ASCs in control and uninfected cohorts. C) IgG and D) IgM PR8‐ specific ASCs. E) IgG and F) IgM total ASCs. G–L) Similarly, mice immunized and boosted with HAd were challenged with 10MID_50_ of PR8 virus at day 14 post‐immunization and measured by ELISPOT at day 2 post‐challenge. M–R) Following intranasal vaccination and boost with PspA or PBS control, or intramuscular prime/boost with Prevnar, half of the mouse cohorts were challenged with 10MID_50_ of PR8 virus at day 14 post‐immunization and measured by ELISPOT at day 2 post‐challenge. The mean (±SD) of 5 mice per cohort are shown, comparing control and challenge cohorts. In a separate experiment, five‐week‐old mice were vaccinated by priming (day 0) with intranasal PspA‐CoPoP/PHAD (PspA) or PBS control, or intramuscular Prevnar‐13 and similarly boosted on day 14. Mice were challenged with 10MID_50_ of PR8 virus or control (PBS) at day 28 post‐immunization. At day 6 post‐viral challenge, all mice received 7.25–10.0 × 10^5^ colony‐forming units (cfu) of *S. pneumoniae* (serotype 3 strain, A66.1) in 50 µL PBS, intranasally. Two days post‐bacterial instillation (PBI), mice were sacrificed and S) lungs homogenized and A66.1 burden determined by titration on blood agar. T) Bodyweight change from the time of PBI (%), and U) clinical score at the time of harvest were also assessed. Data represent mean ± SD. of *n* = 6 for each group (performed in 2 experiments of *n* = 3) except the PBS vaccination/PR8 challenge group experienced 1 death during the 2nd PBI day (*n* = 5). Data from the lung A66.1 burden data were log transformed for normalization before statistical analysis. With (*), (**) indicating Student's paired *t*‐test *P* < 0.05, *P* < 0.01, respectively.

To demonstrate the in vivo effects of viral targeting of adaptive immune responders, cohorts of mice were immunized and boosted with 10 µg of influenza vaccine intranasally or with PBS control before challenge with 10MID_50_ of PR8 at day 7 post‐boost. The influenza‐specific adaptive response was allowed to develop before the influenza challenge to ensure that only direct effects on adaptive responders were observed. On day 2 post‐challenge, lung cells from challenged and control cohorts were isolated, and antigen‐specific and total ASCs were measured by ELISPOT. A decrease in the percentage of H1N1‐specific IgG ASCs (*p* < 0.05) was found after the PR8 challenge (**Figure** **4**A) as well as in total IgG, and IgM ASCs in H1N1 vaccine immunized mice (Figure 4E,F). A decrease in H3N2‐specific IgG ASCs (*p* < 0.05) was also observed (Figure 4A). There is no significant increase in PR8‐responding ASCs to account for this diminishment.

Next, a non‐replicating HAd was used to assess the effect of influenza on HAd‐specific responders to mimic the impact of influenza on recent vaccination and model the impact on respiratory co‐infection.^[^ ^3^, ^5^ ^]^ Cohorts of mice were immunized and boosted intranasally with 10^9^ pfu of HAd before challenge with 10MID_50_ of PR8 at day 7 post‐boost, as before. A reduction (*p* < 0.05) in the percentage of HAd‐specific IgG ASCs was found after the PR8 challenge (Figure 4G), as well as a decrease (*p* < 0.05) in total IgG ASCs (Figure 4K). While declines were trending in both HAd‐specific IgM ASCs (Figure 4H) and total IgM ASCs (Figure 4L), they were not significant. No significant numbers of PR8‐specific ASCs were measured in challenged or control cohorts (Figure 4I,J), so the recruitment of PR8‐specific ASCs does not explain the decrease in HAd‐specific ASCs.

We extended these findings to a bacterial pneumonia model, the most common clinical coinfection,^[^ ^2^, ^11^, ^12^, ^13^, ^14^, ^15^, ^16^, ^39^ ^]^ in which cohorts of mice were immunized and boosted intramuscularly with PREVNAR‐13 vaccine or intranasally with 10 µg pneumococcal surface protein antigen (PspA). A decrease in the frequency of pneumococcal‐specific IgG ASCs (*p* < 0.01) was observed in the cohort immunized with PspA and challenged with PR8 (Figure 4M), while less significant decreases were observed in PREVNAR‐ 13 immunized mice, for PspA‐specific IgM ASCs (Figure 4N), and total IgG and IgM ASCs (Figure 4Q,R). Again, no significant numbers of PR8‐specific ASCs were measured (Figure 4O,P).

In a separate set of experiments, mice were immunized and boosted on day 14 post‐priming intramuscularly with PREVNAR‐13 or intranasally with PspA, and then challenged with 10MID_50_ of PR8 or PBS control on day 28. On day 34 (day 6 post‐PR8 challenge), all mice were challenged with the *S. pneumoniae* strain A66.1 (serotype 3) to simulate human infection with pneumococcus following influenza infection. Morbidity was assessed over the following 48 h, and the mice were sacrificed to determine A66.1 lung titers (Figure 4S). Unvaccinated animals experienced pulmonary bacterial growth whether they had been challenged with PR8 or not, but both PREVNAR‐13‐ and PspA‐vaccinated groups conferred protection following bacterial challenge with significant bacterial clearance. However, this protection was ablated in the PR8‐challenged group resulting in bacterial replication. Assessment of body weight (Figure 4T) and clinical score (Figure 4U) changes confirmed the vaccine protection patterns displayed in the A66.1 lung burden data (Figure 4S). These findings suggest that influenza virus infection compromises pneumococcal vaccine effectiveness in a co‐infection model by infecting and depleting pneumococcal antigen‐specific adaptive immune responders.

## 3 Discussion

Our results demonstrate that in addition to the influenza virus's suppressive effects on the innate immune system,^[^ ^25^, ^29^ ^]^ the virus can also directly suppress adaptive immune responders by infecting and killing activated lymphocytes. This targeting likely results through increased expression of sialic acid on the surface of activated lymphocytes. ASCs were reduced by as much as 50% in infected mouse cohorts or human PBMC samples, which could negatively impact vaccine efficacy and leave patients susceptible to a secondary infection. Though our confirmatory findings are in a mouse model, they are suggestive of a mechanism by which many clinical observations of the immunosuppressive effects of influenza vaccination may be explained and warrant further studies in human challenge models.

Notably, our findings suggest a possible mechanism for previous observations demonstrating a decrease of B and T helper numbers in the lymph nodes during influenza infection,^[^ ^31^ ^]^ as well as evidence of fewer peripheral lymphocytes in humans.^[^ ^40^, ^41^, ^42^ ^]^ Studies have shown diminished adaptive immune responses during influenza to a cell‐free purified protein derivative (PPD) obtained from a human strain of Mycobacterium tuberculosis, candida, mumps virus, and trichophytin were attributed to T cell suppression,^[^ ^43^ ^]^ weakened Ig production, NK functionality^[^ ^44^ ^]^ and/or decreased lymphocyte responsiveness/proliferation.^[^ ^45^, ^46^, ^47^ ^]^ A decrease in circulating lymphocytes, or lymphopenia, is not uncommon in influenza patients.^[^ ^40^, ^41^, ^42^, ^48^, ^49^ ^]^ However, the reasons for this decline were previously unknown, though low levels of influenza viral RNA have been detected in the PBMCs of influenza patients.^[^ ^50^ ^]^ More recently, it has been demonstrated that innate cells and activated B cells were preferentially infected over naïve lymphocytes, although the mechanism is unknown.^[^ ^51^ ^]^ Susceptibility of NK cells as well as B cells to Influenza A virus was also demonstrated via whole lung imaging using NS1‐GFP.^[^ ^52^ ^]^ Similarly, enhanced susceptibility of activated B cells to H5N1 avian influenza was also observed. These investigators reasoned that increased expression of DC‐SIGN on the cell surface might contribute to this and theorized that sialic acid expression might play a role along with the presence of monocytes, though they did not investigate this or any other cell subsets.^[^ ^53^, ^54^ ^]^ Here, we have shown that the immune‐suppressive effect of influenza may occur directly through viral infection and killing of responding immune cells, even in the absence of monocytes – through these and other immune mediators may contribute to additional adaptive cell death in vivo – and that this targeting occurs through increased sialic acid expression. This effect is also observed in innate cells, which may explain previous findings of depleted dendritic cells, macrophages, and NK cells during influenza infection (Figure S3, Supporting Information).^[^ ^19^, ^21^, ^22^, ^23^, ^24^, ^25^, ^26^, ^27^, ^28^, ^29^, ^30^ ^]^ Similar observations were made with measles ‐which has been shown to infect naïve and memory cells, leading to immunological amnesia lasting up to two years.^[^ ^6^, ^55^ ^]^ In the case of influenza, the impact of this immunosuppressive effect is likely limited to the lungs and respiratory tract, based on the localization of the virus itself, although we are yet to test the impact on other compartments – the spleen, bone marrow, and distant lymph nodes. The observed increases in the sialic acid expression on activated cells are part of a normal immune response to infection and do not appear to be specific to influenza infection/vaccination. The increase in cell surface sialic acids is necessary for cell motility and the prevention of cellular adherence. Previous studies have demonstrated sialic acid loss causes clustering and lymphocytosis in humans,^[^ ^56^ ^]^ while upregulation increases cell motility and cancer metastasis.^[^ ^57^, ^58^ ^]^ However, it should be noted that other factors, including other cell surface receptors, may play a role in this targeting as well. While HA has been known to bind sialic acid receptors for cellular entry, NA has been shown to cleave sialic acid from the budding viruses to facilitate their release; however, NA of certain influenza viruses such as N9 have been shown to bind to sialic acids close to enzymatic cleavage site, thus suggesting a potential role along with HA in viral entry.^[^ ^59^, ^60^ ^]^ Sialic acids are merely one of the earliest factors in viral binding and entry, and are targets of both Influenza Hemagglutinin and Neuraminidase, and thus appear to play a significant, early role in this immunosuppressive effect.

Taken together, our findings provide a new mechanism for the reduced efficacy of not only influenza vaccines but also pneumococcal vaccines and the higher incidence of secondary bacterial/viral infections during the influenza season, even in vaccinated or immune individuals. Similarly, co‐circulation of SARS‐CoV‐2^[^ ^61^ ^]^ during the upcoming influenza season may increase disease severity of COVID‐19 even if a vaccine is available.^[^ ^62^ ^]^ Hence, it may be preferable that any vaccine against respiratory pathogens be given well before the influenza season, ensuring that the recipients have high serological and cellular protective responses. Finally, individuals who lack seroprotective immune responses may benefit from passive immunization during influenza seasons.

## 4 Experimental Section

### Mice

BALB/c females were purchased from Jackson Laboratories (Bar Harbor, ME) and used beginning at 5 weeks old and were 8 weeks old at time of challenge. Animals were age‐matched and housed under pathogen‐free conditions. Animal research was conducted under the guidance of the CDC's or the Veterans Administration of Western New York Healthcare System's Institutional Animal Care and Use Committees in an Association for Assessment and Accreditation of Laboratory Animal Care International‐accredited animal facilities.

### Human PBMCs

PBMCs were collected from healthy adult individuals vaccinated with seasonal influenza vaccine during 2013–2014 (H1N1 component A/California/7/2009) and 2018–2019 (H1N1 component A/Michigan/45/2015) influenza seasons at day 7 and 28 post‐vaccination, respectively. PBMCs were thawed, checked for viability, and then cultured in 6‐well plates in complete RPMI [(10% fetal bovine serum, 5% Penicillin/Streptomycin, 2 × 10^−3^ m L‐Glutamine and 50 × 10^−3^ m 2‐ mercaptoethanol)].^[^ ^63^, ^64^ ^]^ The study was conducted as per the protocol approved by the Emory University Institutional Review Board (IRB) (#IRB00045947) and Centers for Disease Control and Prevention‐Reliance IRB (#1652) in compliance with all applicable Federal regulations governing the protection of human subjects.

### Viruses

The Influenza viruses used in this study, A/Puerto Rico/8/34 (PR8); H1N1, and A/California/07/09 (CA09); H1N1,A/Michigan/45/2015;H1N1, A/Victoria/361/2011;H3N2, A/Singapore/INFIMH‐16‐0019/2016;H3N2, B/Massachusetts/2/2012, and B/Phuket/3073/2013 were obtained from the Influenza Reagent Resource (Manassas, VA). NS1‐RFP (A/California/07/2009 backbone) as well as NS1 GFP (A/PR/8/34 backbone) viruses were provided by Dr. Adolfo Garcia‐Sastre, Mount Sinai School of Medicine, New York City, NY47. Viruses were propagated, purified, and stored as described previously.^[^ ^20^ ^]^ A non‐replicating human adenovirus with deletions in E1 and E3 regions (HAd) was provided by Dr. Suresh Mittal, Purdue University, and aliquots stored at −80 °C until use.

### In Vitro PBMC Infection

Two million PBMCs were stimulated with 1 µg mL^−1^ of TLR7/8 agonist R848 in the presence of 10 ng mL^−1^ of IL‐2 for 3 days. At 2 days post‐stimulation, PBMCs were infected with 5 MOI of NS1‐GFP or NS1‐RFP for 1 h at 37 °C in 200 µL of RPMI or treated similarly without adding virus. The cells were cultured overnight at 37 °C in complete RPMI media containing 10% fetal bovine serum (FBS), 5% Penicillin/Streptomycin, 2 × 10^−3^ m L‐Glutamine. On day 3 post‐stimulation, 1 day post‐infection, NS1‐GFP or NS1‐RFP infection was measured via flowcytometry on 3 individual PBMC samples as described under Flow cytometry. At the same time‐point, total and antigen‐specific ASCs were measured by ELISPOT as described under ELISPOTs. All PBMC infections were performed twice, with a total of eight individual PBMCs samples tested.

### Mouse Tissue Isolation

Mice were euthanized, and lung or spleen tissue removed surgically. Briefly, the lungs were processed with the Mouse Lung Dissociation Kit (Miltenyi Biotech) according to the manufacturer's instructions. Dissociated lungs were then separated through 5 mL Lymphoprep buffer (Stemcell) at 900 x *g* for 30 min with centrifuge brakes off. Buffy coat was then removed and washed and resuspended in PBS.

Spleens were ground through a 40 µm cell strainer (Falcon) to disperse cells, treated with Red Blood Cell Lysis Buffer (Sigma) for 5 min, washed, and resuspended in PBS.

### Immunizations and In Vivo Mouse Challenges

In vitro and in vivo experiments were performed with mice immunized and boosted (day 7 post‐ immunization) by intranasal instillation of 10 µg of influenza vaccine, 10^9^ pfu of HAd, 5 MOI of NS1‐GFP or NS1‐RFP, or 50 µL PBS control under 3% isoflurane anesthesia. On day 14 post‐primary vaccination, lungs were collected for in vitro experiments, or mice were challenged with 10MID_50_ of PR8 delivered intranasally in 50 µL performed under 3% isoflurane anesthesia. In another set of in vivo experiments, mice were vaccinated against pneumococcus by priming on day 0 and boosting on day 14 with 50 µL PREVNAR 13 vaccine (diluted 1:3 with PBS) injected into the rear left caudal quadricep (intramuscular) without anesthesia or intranasal pulmonary instillation of 50 µL PBS containing 10 µg pneumococcal surface protein A (PspA) (generously provided by Dr. Blaine Pfeifer, State University of New York at Buffalo, NY) or 2 µg of His‐tagged PspA decorating the exterior of 100 nm cobalt porphyrin‐phospholipid‐phosphorylated hexaacyl disaccharide liposomes (PspA‐CoPoP/PHAD)^[^ ^65^ ^]^ or just PBS vaccination control under 3% isoflurane anesthesia. On day 28, mice were challenged with 10MID_50_ PR8 delivered intranasally in 50 µL performed under 3% isoflurane anesthesia. On day 34 (6 days post‐viral challenge), 7.25–10.0 × 10^5^ cfu of a serotype 3 *S. pneumoniae* strain (A66.1) was delivered in 50 µL PBS, intranasally, under 3% isoflurane anesthesia. Mice were monitored for weight loss and clinical score for two days following bacterial challenge. Clinical score was determined by the addition of single points assessed for each of the following: lethargy, hunched posture, piloerection, abnormal gait, labored breathing, and emaciation (loss of >10% body weight). Those losing >20% body mass were euthanized. Mice were sacrificed 48 h following bacteria challenge and the lungs removed, homogenized using a Bullet Blender tissue homogenizer on setting 8 for 8 min (Next Advance, Troy, NY), and A66.1 titer was performed using trypticase soy agar with 5% sheep blood plates (VWR, Radnor, PA) to determine the lungs’ A66.1 burden. All in vivo mouse experiments were performed on cohorts of at least 3 mice each and repeated 2–3 times.

### In Vitro NS1‐GFP or PR8 Infection and PCR Confirmation

Two million total or 10 000 sorted lung cells were infected with 5MOI of NS1‐GFP (PR8) or wild‐type PR8 for 1 h at 37 °C in 200 µL RPMI or treated similarly without adding virus. Complete media (RPMI with 10% fetal bovine serum, 1% penicillin/streptomycin, 1% HEPES, and 50 × 10^−3^ m 2‐mercaptoethanol) was then added back, and cells were cultured overnight at 37 °C. NS1 expression was measured via flow cytometry. PR8 infection and replication were measured by qPCR, as previously described.^[^ ^20^ ^]^ In brief, RNA was extracted using Trizol, and 2 µg of total RNA was used for cDNA synthesis using Superscript II Reverse Transcriptase (Life Technologies). RT‐PCR was conducted using a Stratagene Q3005 PCR machine and SYBR green (Stratagene, La Jolla, CA) to analyze for the presence of NS1 RNA, using *β*‐actin as a control. All in vitro mouse experiments were performed on samples from at least 3 mice per cohort and repeated 3 times. Lung cells from mice infected with NS1‐GFP in vivo were collected similarly, and NS1 expression was measured by qPCR as described for in vitro infected samples.

### Lectin Blockade

Pooled murine splenocytes were incubated in 200 µL of complete media with dilutions of 1:200, 1:100, or 1:20 Maackia amurensis lectin I (MAA‐1) or II (MAA‐2), or Sambucus nigra lectin (SNA) (Vector Laboratories, Burlingame, CA) for 30 min at 4 °C with 0.5 × 10^−3^ m Ethylenediaminetetraacetic acid (EDTA) added to the culture media to reduce cell clumping. Cells were then washed and incubated with PR8‐FITC for 1 h at 37 °C in 200 µL RPMI, washed again, and incubated overnight in complete media at 37 °C. PR8‐FITC binding was then measured by flow cytometry.

### Flow Cytometry and Sorting

All antibody and lectin staining were performed in staining buffer (BD Biosciences; San Jose, CA) for 30 min at 4 °C in the dark. Murine antibodies used included: Live/Dead Fixable Blue Dead Cell Stain (Invitrogen Carlsbad, CA), B22 PE‐Cy7 (RA3‐6B2, Biolegend, San Diego, CA), CD138 APC (281‐2, Biolegend), CD38 Alexa700 (HIT2, eBioscience, San Diego, CA), CD69 PE (H1.2F3, Biolegend), GL7 Pacific Blue (GL7, Biolegend), CD3 BV6 (SP34‐2, BD), CD4 A700 (RPA‐T4, Biolegend), CD8 Pacific Blue (53‐6.7, BD), CD44 PE‐Cy7(IM7, BD), CD62L APC (MEL‐14, Biolegend), and CD49b PE (DX‐5, Biolegend). The innate cell panel in Figure S4 (Supporting Information) also includes CD11c APC‐Cy7 (N418, eBioscience), CD11b PacificBlue (M1/70.15, eBioscience), M2 Alexa700 (eBioscience), CD45 AmCyan (30‐F11, Biolegend), Ly6G APC (1A8, Biolegend), CD64 PE (X54‐5/7.1, Biolegend), and CD24 BV605 (M1/69, Biolegend). Human antibodies used included: Live/Dead Fixable Blue Dead Cell Stain (Invitrogen), CD PerCP (UCHT1, Biolegend), CD20 APC‐Cy7 (2H7, Biolegend), CD69 BV605 (H1.2F3, Biolegend), CD27 BV650 (M‐T271, BD), CD19 PE (HIB19, BD), CD38 PE‐Cy7 (HIT2, Biolegend), CD4 PE‐Cy5 (RPA‐T4, BD), IgD Pacific Blue (11‐26c.2a, Biolegend), CD14 Alexa700 (63, D3, Biolegend), CD56 APC (HCD56, Biolegend), and NS1‐GFP. Lectins included MAA‐1, MAA‐2, and SNA, either FITC‐conjugated or biotinylated (Vector Laboratories). Death was measured by 7AAD (BD) and Annexin V (BD) staining. Staining was analyzed on the BD LSRFortessa, and B, and T cell subsets were sorted on the BD FACSAria. Gating strategies are shown in Figures S1–S2 (Supporting Information). To gate between low and high‐expression populations where populations were not fully distinct (such as CD44), a histogram of total antibody expression was used to determine the boundary line between the two population peaks, set at the lowest trough.

### ELISPOTs

96 well plates were coated with 100 HA of A/California/07/2009;H1N1, A/Michigan/45/2015;H1N1,A/Victoria/361/2011;H3N2, A/Singapore/INFIMH‐160019/2016;H3N2, B/Massachusetts/2/2012, B/Phuket/3073/2013, A/PR/8/34, 10 µg mL^−1^ Rubeola Measles antigen (Meridian Life Science), or 10 µg mL^−1^ varicella zoster virus antigen (Meridian Life Science10^6^ pfu mL^−1^ HAd, 5 µg mL^−1^ PspA, or 5 µg mL^−1^ IgG or IgM (Southern Biotech). All plates were allowed to coat overnight at 4 °C and the next day blocked with complete media. Cultured human PBMCs or lung cells isolated from immunized mice were serially diluted in complete media and incubated overnight at 37 °C. The plates were then treated with either anti‐IgG‐ or ‐IgM‐biotin (Southern Biotech), followed by incubation with streptavidin‐alkaline phosphatase (Southern Biotech). Plates were then developed with the Vector Blue Alkaline Phosphatase Substrate Kit (Vector Laboratories) until spots developed and spots counted and analyzed with CTL immunospot software.

### Statistical Analysis

Statistical significance was verified by either the Student's *t*‐test (two‐tailed), paired or unpaired as indicated and appropriate to the experimental design, or One‐way ANOVA with Dunnett's Multiple Comparison where three or more groups were compared. For the in vivo vaccination and concurrent influenza‐ and pneumococcal‐challenge experiment (Figure 4S–U), following log_10_ transformation of the A66.1 lung burden data for normalization, a one‐way ANOVA with Dunnett's Multiple Comparison to a Control post‐ hoc test was performed for both PBS control vaccination groups. Besides, unpaired, two‐tailed Student's *t*‐tests were performed comparing the two virus challenges (control and PR8) within each vaccination group, except for the clinical score, which used a Wilcoxon Signed Rank test (due to values = 0 for all the vaccinated, control challenge mice). The mean and standard deviation (SD) are shown for all analyses. Data was normalized only where indicated – for infected PCR samples normalized to naïve levels set at 1, and in 7AAD/Annexin‐5 analyses in comparison to the matched uninfected subset – and all outliers included in the final analysis. Statistical analyses were performed with GraphPad Prism version 6.00 (La Jolla, CA, www.graphpad.com).

## Conflict of Interest

The authors declare no conflict of interest.

## Author Contributions

S.S. conceived the idea, participated in the planning, supervision, and execution of the experiments that led to the present manuscript. C.D.B. participated in the planning and execution of all experiments (except for the in vivo pneumococcal challenge performed at SUNY Buffalo) and wrote this manuscript with the help of S.S. Z.E. assessed the effect of influenza virus on human PBMC in vitro, with the assistance of M.M. for gating strategies and flow cytometric analysis. W.C. assessed innate cell subsets for levels of sialic acid expression and influenza infection, and assisted C.D.B. with mouse infections, immunizations, and tissue collections, along with W.M. P.R. performed qPCRs to confirm viral replication in infected cells. A.K. offered guidance for in vivo and in vitro fluorescently labeled influenza infections and assisted with microscopy to ensure immune cell infection. S.A. provided technical advice for T cell panels. S.K.M., A.G.‐S., B.A.P., and J.F.L. provided reagents crucial for carrying out the studies. B.A.D. performed in vivo studies with influenza, and pneumococcal challenge, and P.K. oversaw the investigations with pneumococcal challenges and provided guidance. S.G. oversaw all mouse experiments and provided guidance.

## Supporting information

Supporting Information

## Data Availability

All data are presented as figures. Raw data can be provided if needed.
