# Supplementary material for: Influenza Virus Infects and Depletes Activated Adaptive Immune Responders
Source: Adv Sci (Weinh). 2021 Jun 30;8(16):2100693. doi: 10.1002/advs.202100693 (PMC8373117; doi:10.1002/advs.202100693)

© 2021 Wiley-VCH GmbH

ADVANCED SCIENCE Open Access

Supporting Information

for *Adv. Sci.*, DOI: 10.1002/advs. advs202100693

Influenza virus infects and depletes activated adaptive immune responders

Caitlin D. Bohannon[1,2], Zachary Ende[1,2], Weiping Cao[1], Wadzanai P. Mboko[1,3], Priya Ranjan[1], Amrita Kumar[1], Margarita Mishina[1], Samuel Amoah[1], Shivaprakash Gangappa[1], Suresh K. Mittal[3], Jonathan F. Lovell[4], Adolfo García-Sastre[5,6,7,8], Blaine A. Pfeifer[9], Bruce A. Davidson[10,11,12], Paul Knight[10], and Suryaprakash Sambhara[1]**

Supplementary Figures:

Supplementary Figure 1: Gating strategy in human PBMCs. (A) B cell gating. (B) T cell gating.

Supplementary Figure 2: Gating strategy in mice. (A) B cell gating. (B) T cell gating.

Supplementary Figure 3: Influenza virus preferentially targets mouse innate cells expressing high levels of α2,3 sialic acids. (A) Representative lectin binding and NS1-GFP expression of interstitial and alveolar macrophages and CD11b+/- dendritic cells before *in vivo* infection with PR8, (B) at day 1 p.i., (C) day 3 p.i., and (D) day 7 p.i. Pooled lung cells from 3-4 mice were used and the experiment was repeated at least twice.

Supplementary Figure 1

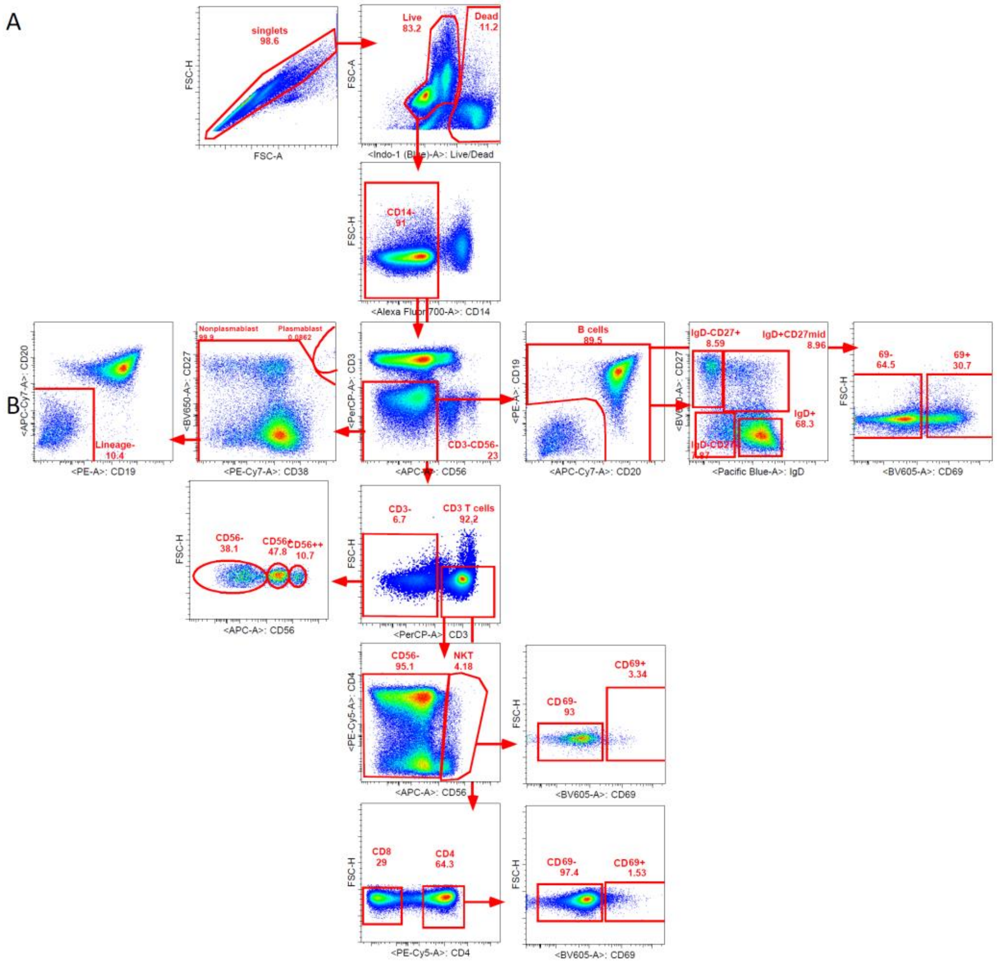

Supplementary Figure 2

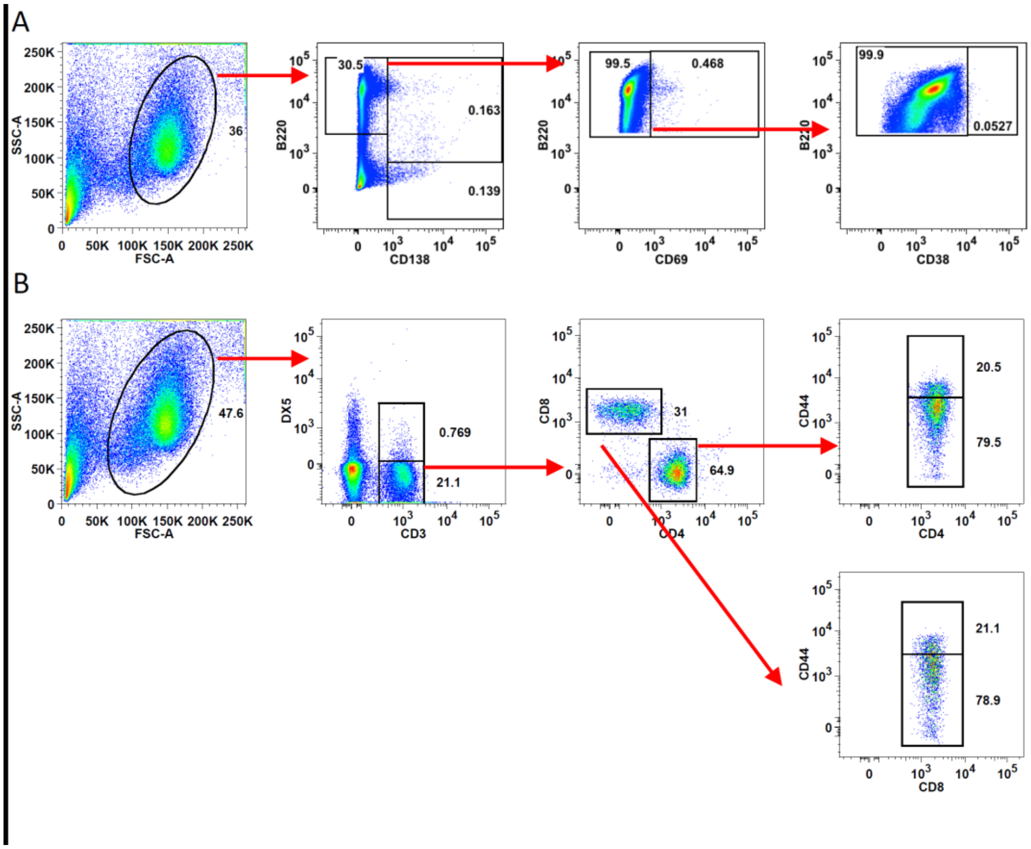

Supplementary Figure 3

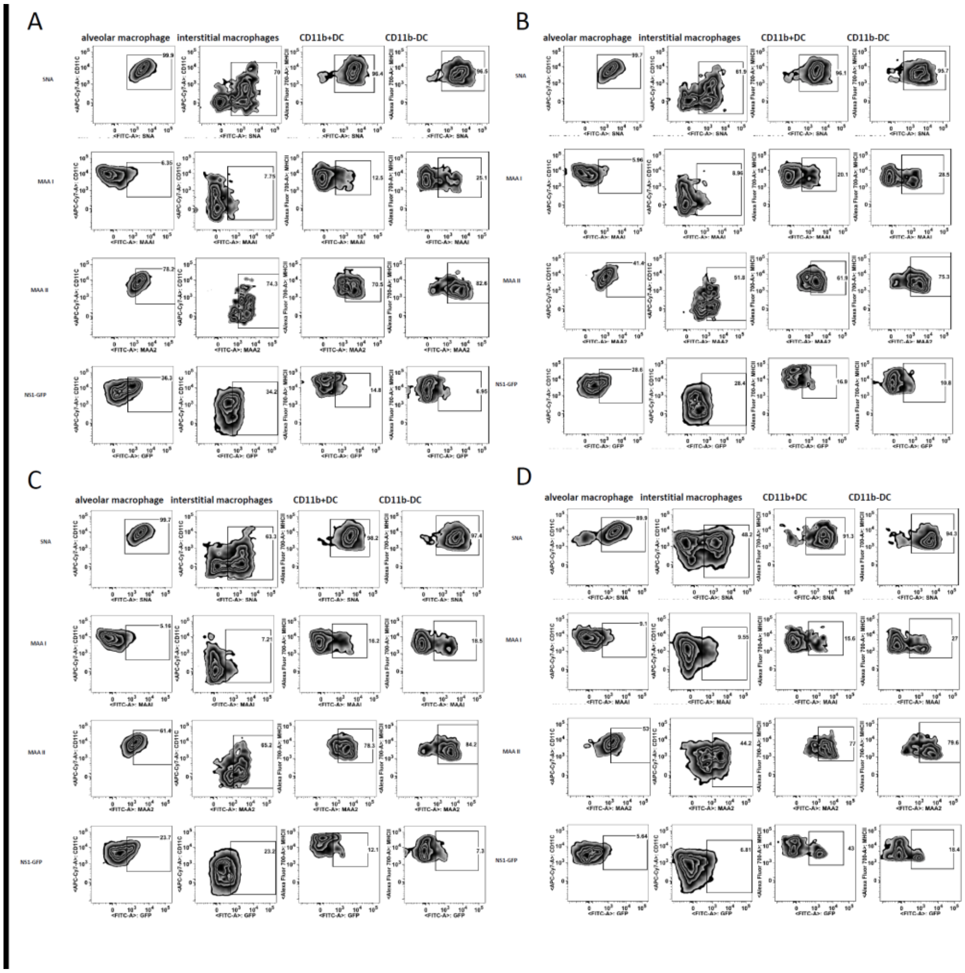

Supplement: Supplementary file 1 — Supporting Information [file ADVS-8-2100693-s001.pdf]
